# DNA damage-induced metaphase I arrest is mediated by the spindle assembly checkpoint and maternal age

**DOI:** 10.1038/ncomms9706

**Published:** 2015-11-02

**Authors:** Petros Marangos, Michelle Stevense, Konstantina Niaka, Michaela Lagoudaki, Ibtissem Nabti, Rolf Jessberger, John Carroll

**Affiliations:** 1 Department of Cell and Developmental Biology, Division of Biosciences, UCL, Gower Street, London WC1E 6BT, UK; 2 Department of Biological Applications and Technology, University of Ioannina, 45110 Ioannina, Greece; 3 Institute of Physiological Chemistry, Faculty of Medicine Carl Gustav Carus, Dresden University of Technology, Fiedlerstrasse 42, MTZ, 01307 Dresden, Germany; 4 Development and Stem Cells Program, Monash Biomedicine Discovery Institute and Department of Anatomy and Developmental Biology, Monash University, Melbourne, Victoria 3800, Australia

## Abstract

In mammalian oocytes DNA damage can cause chromosomal abnormalities that potentially lead to infertility and developmental disorders. However, there is little known about the response of oocytes to DNA damage. Here we find that oocytes with DNA damage arrest at metaphase of the first meiosis (MI). The MI arrest is induced by the spindle assembly checkpoint (SAC) because inhibiting the SAC overrides the DNA damage-induced MI arrest. Furthermore, this MI checkpoint is compromised in oocytes from aged mice. These data lead us to propose that the SAC is a major gatekeeper preventing the progression of oocytes harbouring DNA damage. The SAC therefore acts to integrate protection against both aneuploidy and DNA damage by preventing production of abnormal mature oocytes and subsequent embryos. Finally, we suggest escaping this DNA damage checkpoint in maternal ageing may be one of the causes of increased chromosome anomalies in oocytes and embryos from older mothers.

## Abstract (web summary)

DNA damage in mammalian oocytes can lead to infertility and developmental disorders. Here Marangos *et al.* show that the spindle assembly checkpoint responds to DNA damage by arresting oocytes in metaphase I, and this checkpoint becomes compromised as mice age.

## Graphical abstract

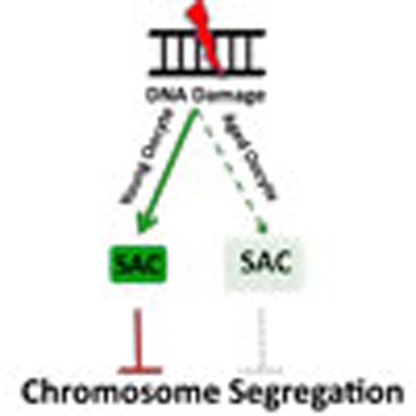

DNA damage is a constant threat to cells and its harmful effects accumulate with age^1^,^2^. Cells with long lifespans are therefore particularly prone to accumulating DNA damage. One such population is the mammalian oocyte, which remains arrested at G2/prophase in the ovary for the entire reproductive lifespan that can be 40 years in humans.

The resting pool of primordial follicles is particularly sensitive and DNA damage leads to apoptosis via the Tap63 pathway^3^,^4^,^5^,^6^. There is much less known about DNA damage in oocytes at later stages of development when the Tap63 pathway is inactive. We recently showed that fully grown oocytes are not capable of establishing a robust G2/prophase checkpoint in response to DNA damage. The induction of double-strand breaks (DSBs) by genotoxic agents such as Etoposide and Doxorubicin does not arrest mouse oocytes in G2/prophase but, instead, allows entry into the first meiotic M-phase (MI)^7^. Etoposide and Doxorubicin are topoisomerase II poisons that form stable complexes with the DNA and topoisomerase II, a DNA unwinding enzyme and therefore prevent re-ligation of DNA strands, leading to DNA DSBs^8^. Besides DSBs, other DNA lesions, such as interstrand crosslinks, also fail to induce the launch of a G2/prophase checkpoint in mouse oocytes^9^. Therefore, a checkpoint during the meiotic M-phase may raise the only significant obstacle to DNA damage entering embryonic development.

Interestingly, in oocytes, maternal age-related genomic instability has been linked to meiotic M-phase chromosome segregation errors such that over 60% of oocytes in women over the age of 40 have major chromosomal problems^10^,^11^. To date, the cause of increased aneuploidy and chromosomal anomalies in oocytes from aged mice has been attributed mainly to the loss of Cohesin, the protein complex responsible for holding chromosomes together^12^,^13^,^14^,^15^ but also to the failure to correct erroneous microtubule–kinetochore interactions^16^ and to a leaky SAC^11^,^17^,^18^.

In oocytes undergoing meiotic M-phase, the DNA damage response and how it is impacted by maternal age is poorly understood. Here, we report that exposing oocytes to genotoxic agents causes SAC-dependent M-phase arrest at MI. Nevertheless, we find that, in oocytes from mice of advanced maternal age, DNA damage-induced MI arrest is compromised and this appears to be due to disruption of the SAC. These results highlight the importance of the SAC for preventing DNA damage-induced genomic instability in oocytes and identify DNA damage as a potential cause for age-related infertility.

## Results and Discussion

### DNA damage in oocytes leads to MI arrest

**Figure 1:**
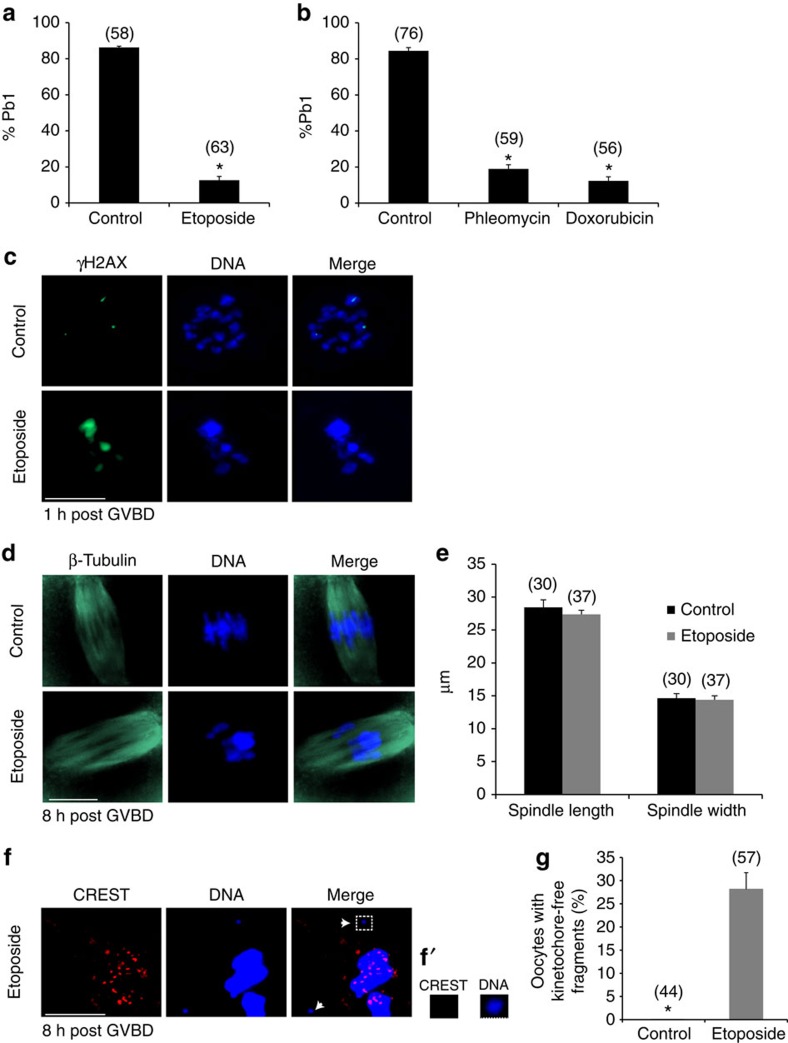
DNA damage induces MI arrest in mouse oocytes. (**a**) Oocytes were treated with Etoposide (100 μg ml^−1^), (**b**) Phleomycin (10 μg ml^−1^) or Doxorubicin (20 μM) for 1 h during the GV stage and released from IBMX. First polar body (Pb1) extrusion was scored 18 h after release from IBMX and oocytes continued to be monitored for Pb1 extrusion until at least 24 h from IBMX release. The total number of oocytes examined is shown in parentheses. *n*≥3 experiments. (**c**) DNA damage induced at GV stage persists to MI. Representative images of γH2AX and DNA labelling in control and Etoposide-treated oocytes fixed 1 h post GVBD. Total ≥20 oocytes/group from three independent experiments. (**d**,**e**) Spindle formation is grossly normal in control and Etoposide-treated oocytes. (**d**) Representative images of microtubule and DNA organization in MI oocytes exposed to DNA damage fixed 8 h post GVBD. (**e**) The spindle length and width of oocytes represented in **d** are the same under control and Etoposide conditions; *n*=3 experiments. (**f**,**g**) Kinetochore-free DNA fragments are generated by Etoposide treatment. (**f**) Representative images from immunostained Etoposide-treated MI oocytes fixed 8 h post GVBD. CREST is used to label kinetochores. Arrowheads: Etoposide-induced kinetochore-free DNA fragments. White box: DNA fragment shown in higher magnification in **f'**. Hoechst 33342 was used for DNA staining, as in **c** and **d**. (**g**) Oocytes from five experiments performed under the conditions of **f** were examined for the presence of kinetochore-free DNA fragments. Approximately 30% of Etoposide-treated oocytes possess at least one kinetochore-free DNA fragment as in **f**. Solid bars, 10 μm. Dashed bar, 2 μm. Data are represented as mean. Error bars show s.e.m.'s; *P<0.0001; unpaired *t*-test.

In the absence of a robust G2/M DNA damage checkpoint the potential to create mature eggs capable of undergoing fertilization carries the risk of creating embryos with chromosomal abnormalities. We therefore set out to investigate the fate of oocytes carrying DNA damage into the first meiotic division. DNA damage was induced in fully grown, prophase-arrested germinal vesicle (GV) stage oocytes using well characterized genotoxic agents that cause DSBs, namely Etoposide (100 μg ml^−1^, 1 h), Phleomycin (10 μg ml^−1^, 1 h) and Doxorubicin (20 μM, 1 h)^7^,^9^,^19^. The oocytes were then monitored for progression through meiosis I. Consistent with previous studies^7^,^9^, oocytes with DNA damage do not show a G2/prophase checkpoint, irrespective of the agent used (Fig. 1a,b). However, our data show that <20% of oocytes treated with Etoposide, Phleomycin or Doxorubicin progress through meiosis I and extrude a first polar body (Pb1), compared with more than 80% of control oocytes (Fig. 1a,b). Since all agents cause a similar MI arrest phenotype, Etoposide will be used to induce DNA damage in all subsequent experiments. We have confirmed that the DNA damage induced in prophase-arrested oocytes persists through to MI as demonstrated by γH2AX labelling (Fig. 1c).

To investigate the target of DNA damage in causing MI arrest we analysed the spindle and chromosome configuration in Etoposide-treated oocytes. Analysis of spindle size and shape shows that DSBs do not have any significant effect on the spindle (Fig. 1d,e). However, Etoposide treatment does cause chromatin aggregations and reduced chromosome alignment at the metaphase plate (Fig. 1c,d and Supplementary Fig. 1). We also find evidence of kinetochore-free chromosome fragments in nearly 30% of Etoposide-treated oocytes (Fig. 1f,g). If these oocytes progressed through MI, the fragments would not participate in chromosome segregation, thereby, causing the generation of oocytes with chromosomal abnormalities and altered DNA content. These results show that oocytes respond to DSBs by establishing an MI arrest. This DNA damage MI checkpoint therefore provides a mechanism of avoiding the production of embryos that show chromosomal abnormalities and potential genomic instability.

### The SAC is active in DNA-damaged MI-arrested oocytes

**Figure 2:**
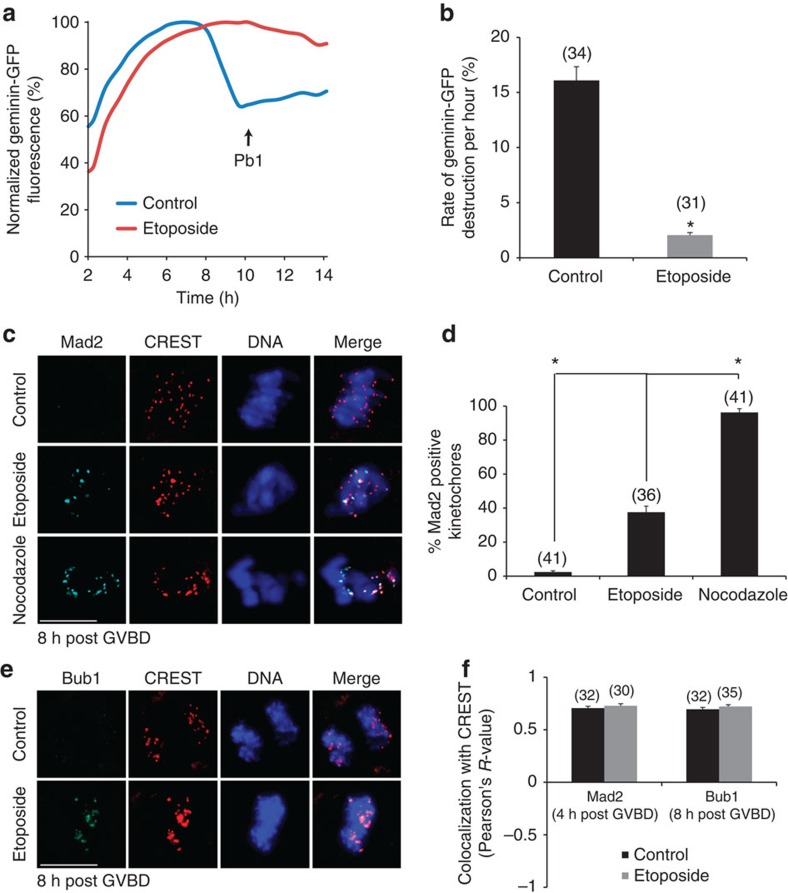
The SAC is active during MI arrest in response to DNA damage. (**a**,**b**) The APC/C is not activated in DNA-damaged oocytes. (**a**) Representative fluorescent traces of oocytes micro-injected with Geminin-GFP at the GV stage, incubated in the presence or absence (Control) of Etoposide for 1 h during the GV stage and then released from IBMX. Normalization was performed by using the formula *F* × 100/*F*_max_. Pb1, first polar body. (**b**) The rate of destruction of Geminin-GFP in oocytes from **a** was significantly higher in controls at the time of the metaphase to anaphase transition in MI. At the same time, Etoposide-treated oocytes remain arrested at prometaphase since they hardly show any level of Geminin-GFP destruction. (**c**,**d**) Etoposide-treated oocytes arrested in MI are Mad2 positive. (**c**) Representative *z*-projection images of immunostaining for CREST and Mad2 in non-treated controls and oocytes treated with Etoposide or Nocodazole (100 nM). Oocytes were fixed 8 h post GVBD. The colocalization of CREST with Mad2 shows that the SAC is only active at the kinetochore region. (**d**) Proportion of Mad2 positive kinetochores 8 h post GVBD. Data analysed from experiments shown in **c**. The total number of cells measured is shown in parentheses; *n*≥3 experiments. (**e**) Etoposide-treated oocytes arrested in MI are Bub1 positive. Representative *z*-projection images of immunostaining for CREST and Bub1 in non-treated controls and oocytes treated with Etoposide. Oocytes were fixed 8 h post GVBD. The control oocyte shown is undergoing anaphase at which point Bub1 staining is lost from the kinetochores. At the same time, Etoposide-treated oocytes remain arrested at MI with high Bub1 staining. In all, ≥20 oocytes were examined per group; *n*=3 experiments. (**f**) SAC components are only present at the kinetochore region of DNA-damaged MI oocytes. Prometaphase control and Etoposide-treated oocytes were fixed at 4 and 8 h post GVBD for identifying the level of co-staining of CREST with Mad2 or Bub1. The 4 h time point was chosen for Mad2-CREST colocalization because Mad2 shows very low staining at 8 h in controls. CREST and SAC components are colocalized at the same levels in control and Etoposide conditions. Scale bars, 10 μm. Data are represented as mean. Error bars show s.e.m.'s; *P<0.0001; unpaired *t*-test.

To examine whether MI arrest occurred before activation of the anaphase promoting complex/cyclosome (APC/C), we expressed Geminin-GFP (green fluorescent protein) in oocytes and monitored its destruction in control and Etoposide-treated oocytes. Geminin has the advantage in this assay that, unlike other APC substrates, it does not have a role in meiotic progression and therefore is less likely to interfere with meiosis^20^. Etoposide-treated oocytes progressing into MI show stable levels of Geminin-GFP for a number of hours and fail to extrude Pb1 (Fig. 2a,b). In contrast, control oocytes show rapid degradation of Geminin-GFP by 8 h post germinal vesicle breakdown (GVBD; Fig. 2a,b) and undergo Pb1 extrusion. Cyclin B1 degradation has also been shown to be inhibited in oocytes with Bleomycin-induced DNA damage^19^. These results show that DSBs cause prometaphase/metaphase arrest at MI.

Failure to activate the APC/C implicates a role for the SAC in the DNA damage-induced MI arrest. The SAC operates physiologically to sequester Cdc20 and inhibit the APC/C so that anaphase onset is delayed until all chromosomes are under tension on the M-phase spindle. SAC activity is regulated by a protein complex that includes Mad2, Bub1, BubR1 and Mps1, which accumulate on unattached kinetochores^21^,^22^,^23^,^24^,^25^,^26^. SAC function is regulated at two levels: the level of kinetochore–microtubule attachment, which is monitored by factors such as Mad2 and the level of kinetochore tension, which is controlled by factors such as Bub1 and BubR1 (refs 24, 25, 26). To examine if the SAC is responsible for DNA damage-induced MI arrest, we used immunofluorescence to determine whether Mad2 and Bub1 remain on the kinetochores. In control conditions, Mad2 is removed from kinetochores by 8 h post GVBD^22^ and we find that only 3% of the kinetochore population shows any detectable Mad2 staining (Fig. 2c,d). However, after DNA damage, an average of 38% of kinetochores in each Etoposide-treated oocyte are Mad2 positive (Fig. 2c,d). Similar results are found in Phleomycin-treated oocytes where 34% of kinetochores are decorated with Mad2 (Supplementary Fig. 2). Unlike Nocodazole-treated cells where practically all the kinetochores are Mad2 positive, in Etoposide-treated cells more than 60% of kinetochores have obtained kinetochore–microtubule attachment (Fig. 2c,d). Similarly, in Etoposide-treated oocytes, Bub1 is strongly localized at kinetochores at the time when control oocytes enter anaphase showing no detectable Bub1 kinetochore accumulation (Fig. 2e). Although it has been reported in some mitotic cells that SAC proteins can be found at sites of DNA damage^27^,^28^, we find that SAC proteins are exclusively localized to kinetochores (Fig. 2c–e). This is verified by image analysis showing that the level of colocalization of Mad2 or Bub1 with CREST (calcinosis, Raynaud's phenomenon, esophageal dysmotility, sclerodactyly, telangiectasia) is the same regardless of whether or not DSBs have been induced (Fig. 2f). This finding implies that, in oocytes, M-phase arrest in response to DNA damage is a result of the kinetochores sensing peri-centromeric damage or of inappropriate kinetochore–microtubule interactions/tension due to DNA damage and not of SAC activation at the site of the DSB. Thus, oocytes with DNA damage fail to activate the APC/C and show an accumulation of SAC proteins at kinetochores, strongly implicating the SAC in the DNA damage-induced MI arrest.

### The SAC establishes DNA damage-induced MI arrest

**Figure 3:**
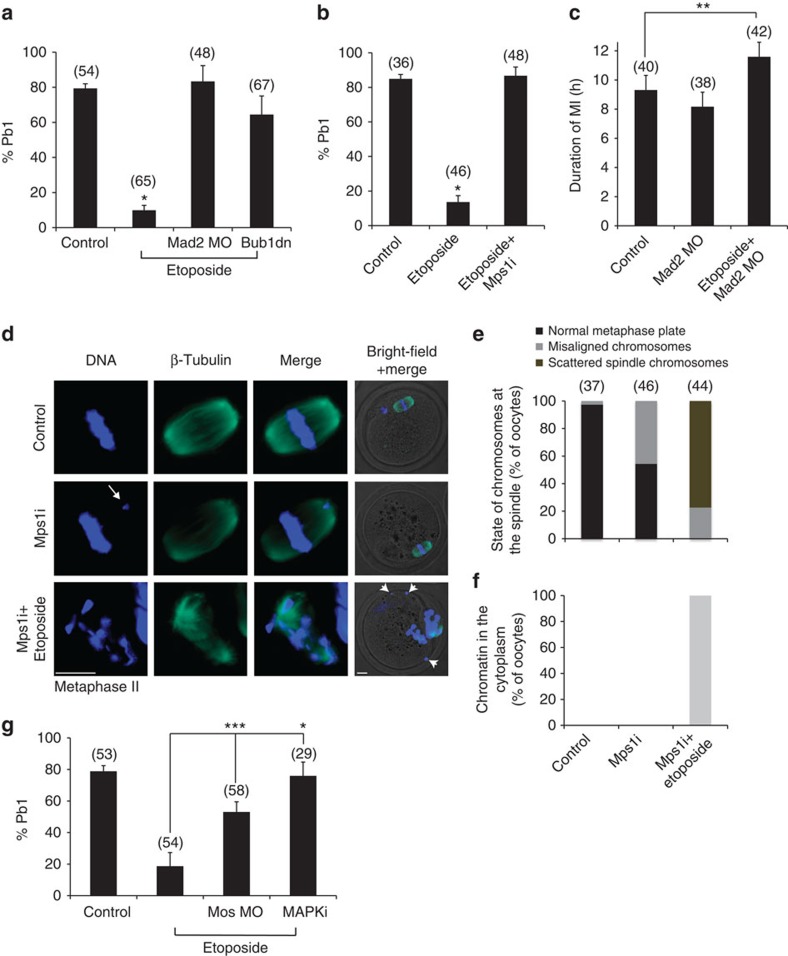
Inactivation of the SAC allows DNA-damaged oocytes to exit MI. (**a**,**b**) Oocytes were treated with Etoposide as previously described and three independent approaches were taken to inhibit the SAC. Depletion of Mad2 (Mad2 MO), injection of Bub1dn or inhibition of Mps1 (Mps1i) all alleviate the MI arrest induced by DNA damage. Pb1 extrusion was scored 18 h after release from IBMX and oocytes continued to be monitored for Pb1 extrusion until at least 24 h from IBMX release. (**c**) Oocytes were monitored during oocyte maturation to determine the duration of MI, from GVBD to Pb1 extrusion. MI is prolonged under conditions of DNA damage when the SAC is suppressed; *n*≥3 experiments for **a**–**c**. (**d**–**f**) Mps1 inhibition in DNA-damaged oocytes leads to gross abnormalities in chromosome configuration at MII. (**d**) Representative images of MII oocytes fixed 14 h post GVBD for β-tubulin immunolabelling and stained with Hoechst 33342 to determine the state of the spindle and chromatin, respectively, following DNA damage and treatment with Mps1i; *n*=3 experiments. (**e**) Analysis of MII oocytes from **d** shows that, unlike control and Mps1i alone-treated oocytes, oocytes exposed to both Etoposide and Mps1i possess scattered chromatin within the spindle with no visible metaphase plate (see representative image in **d**). Misaligned chromosomes are considered the ones lying fully outside the area of a clear metaphase plate (6 μm × 12 μm) (see representative image for Mps1i alone-treated cells in **d**). (**f**) Oocytes treated with both Etoposide and Mps1i from **d** bear chromatin that has been lost from the spindle and is instead found in the cytoplasm. Arrow: misaligned chromosome; arrowhead: cytoplasmic chromatin. Scale bar, 10 μm. (**g**) MAPK contributes to DNA-induced MI arrest. Inhibition of MAPK was achieved by injection of Mos morpholinos (Mos MO) into GV-stage oocytes or by applying a MAPK kinase inhibitor (MAPKi). Pb1 extrusion in control and Etoposide-treated oocytes was scored 18 h after release from IBMX; *n*=3 experiments. The total number of oocytes in each experiment is shown in parentheses. Data are represented as mean. Error bars show s.e.m.'s; *P<0.0001; **P<0.01; ***P<0.001; unpaired *t*-tests.

To identify the importance of the SAC in regulating the MI response to DNA damage we have suppressed the SAC using three independent approaches and examined the effects on MI progression under conditions of DNA damage. We find that depletion of Mad2 by the use of a Mad2 morpholino (MO), inhibition of Bub1 by a dominant-negative form of the protein (Bub1dn) or of Mps1 by the use of the specific inhibitor AZ3146 (Mps1i), allows oocytes with DNA damage to complete MI and extrude a Pb1 at rates of 83%, 64% and 85%, respectively (Fig. 3a,b). We verified that Mad2 MO and Mps1i cause SAC inactivation by determining that Mad2 accumulation in kinetochores is largely diminished in the treated oocytes (Supplementary Fig. 3a,b). Similarly, Bub1 dn injection leads to a more than 60% reduction in BubR1 levels (Supplementary Fig. 3c,d). The rate of Pb1 formation when the SAC is perturbed is similar to control oocytes not exposed to any DNA damage (79–84%) and much higher than in oocytes with DNA damage and an intact SAC (∼10%) (Fig. 3a,b). Although, these studies show that inhibition of the SAC allowed oocytes with DNA damage to progress through MI, we could not rule out an effect of DNA damage on the timing of meiotic progression. To address this we monitored Pb1 formation in Mad2-depleted oocytes and found that DNA damage led to delayed completion of MI relative to controls (∼12 h compared with ∼9 h post GVBD, respectively; Fig. 3c). Mad2-depletion was clearly effective in these experiments because, as expected, Mad2 MO-injected control oocytes progressed through MI faster than controls with an intact SAC (Fig. 3c). Oocytes with DNA damage that progressed through to meiosis II (MII) in the presence of Mps1i showed spindle disruption and chromosome scattering with evidence of chromatin present in the cytoplasm completely detached from the spindle. This was not seen in untreated control oocytes and Mps1i-treated control oocytes only showed mild chromosomal misalignment in 45% of oocytes (Fig. 3d–f). These three different approaches provide consistent data that demonstrate that SAC activity is necessary for DNA damage-induced MI arrest and that DNA damage may induce enhanced SAC activity, or SAC-dependent pathways that lead to a delay in progression through MI.

The Mos/MAPK pathway in oocytes is activated just after GVBD and has a number of roles in meiosis including a well-established role in maintenance of MII arrest^29^,^30^. Recent data have shown a role for MAPK in regulating the meiotic SAC^31^, suggesting it may also be important in ensuring oocytes with DNA damage do not progress. We targeted the Mos/MAPK pathway by depleting Mos through a well characterized Mos-specific MO^31^ and by inhibiting the MAPK activating kinase, MEK, with the pharmacological inhibitor UO126 (MAPKi) in Etoposide-treated oocytes. We find that Mos depletion or MAPK inhibition ablate the DNA damage-induced MI checkpoint leading to 53% and 76% Pb1 extrusion, respectively (Fig. 3g). Therefore, the Mos/MAPK pathway participates in the establishment of MI arrest in the presence of damaged DNA. The role of the Mos/MAPK pathway, most possibly, involves the enhancement of SAC activity since MAPK is important for the kinetochore localization of SAC proteins^31^,^32^,^33^. Besides the importance for meiotic processes, the identification of the Mos/MAPK pathway as a regulator of mammalian M-phase checkpoint function may shed light on its action in mitosis and specifically, in Mos-positive tumours^34^,^35^.

We then examined if a traditional DNA damage response (DDR) intersects with the SAC to promote and maintain an MI arrest. The G2 DDR pathway depends on ataxia telangiectasia mutated (ATM) kinase-dependent cell cycle arrest^36^,^37^. By using a specific well-characterized pharmacological inhibitor against ATM (ATMi, Calbiochem)^7^ we find that ATM inhibition does not prevent MI arrest of DNA-damaged oocytes (∼10% Pb1 extrusion; Supplementary Fig. 4a). Furthermore, ATM inhibition does not prevent SAC components, such as Mad2, from accumulating on the kinetochores of DNA-damaged MI oocytes (Supplementary Fig. 4b,c). Therefore, an ATM-dependent DDR mechanism does not participate in SAC establishment or maintenance in DNA-damaged oocytes.

### MI arrest is compromised in DNA damaged aged oocytes

**Figure 4:**
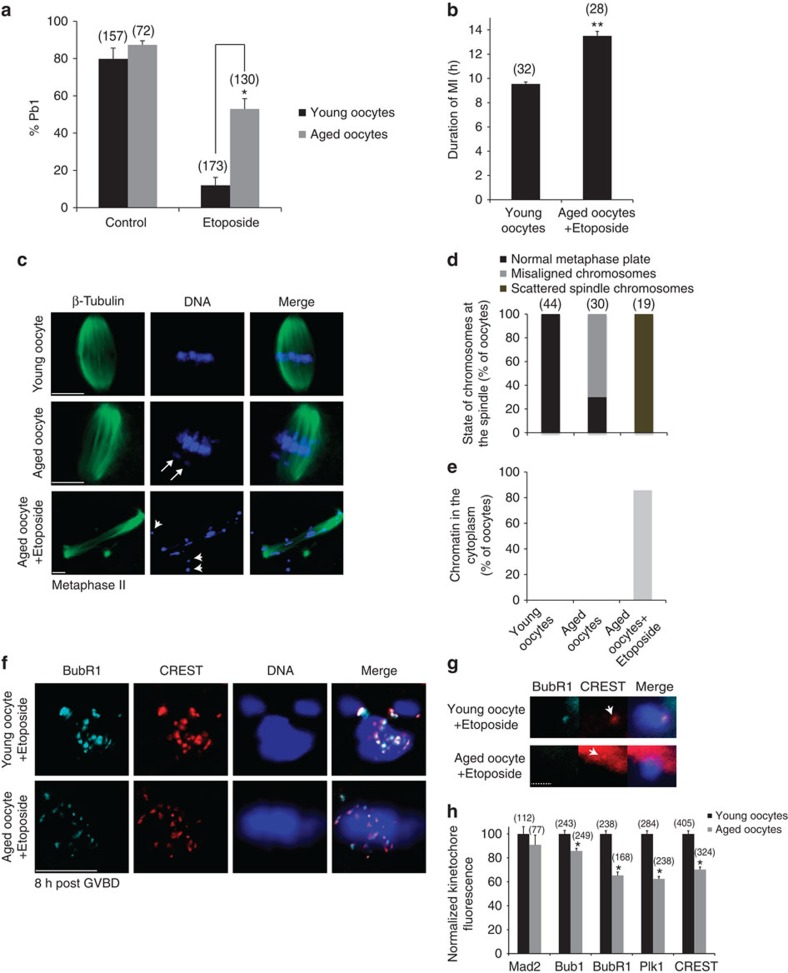
Aged oocytes carrying DSBs possess a weaker SAC than young oocytes. (**a**) DNA-damaged aged oocytes complete MI and extrude a Pb1. Young and aged oocytes were monitored for Pb1 extrusion 18 h after release from IBMX. Etoposide was used at the GV stage; *n*=11 experiments. (**b**) Young and aged oocytes were monitored during oocyte maturation to determine the duration of MI. MI is prolonged under conditions of DNA damage in aged oocytes compared with undamaged young oocytes; *n*=3 experiments. (**c**–**e**) Etoposide-treated aged oocytes reaching the MII stage possess severely abnormal chromatin configuration and spindle structures. (**c**) Representative images of MII oocytes fixed 18 h post GVBD and used for immunostaining with β-tubulin and DNA staining with Hoechst 33342 to determine spindle structures and the state of the chromatin, respectively, following DNA damage in aged oocytes at the GV stage. (**d**,**e**) Analysis of MII oocytes from **c** was performed as in Fig. 3e,f. Etoposide-treated aged MII oocytes are characterized by scattered chromosomes within an expanded spindle structure and by cytoplasmic chromatin; *n*=3 experiments. Arrow: misaligned chromosome; arrowhead: cytoplasmic chromatin. The total number of oocytes examined in **a**–**e** is shown in parentheses. (**f**) Aged oocytes show lower BubR1 accumulation at the kinetochores following DNA damage. Representative *z*-projection images of immunostaining for CREST and BubR1 in young and aged oocytes treated with Etoposide. Oocytes were fixed 8 h post GVBD. (**g**) BubR1 accumulation in bivalents of young and aged oocytes. The arrow shows the position of the kinetochore. Note the absence of BubR1 and the low CREST fluorescence on the kinetochore region of the aged oocyte bivalent; *n*=2 experiments. (**h**) Aged oocytes show lower accumulation of kinetochore components following DNA damage. Quantification of Mad2, Bub1, BubR1, Plk1 and CREST kinetochore fluorescence from experiments (*n*=2) performed under the conditions shown in **f**. The number of kinetochores measured is shown in parentheses. Data are represented as mean. Error bars show s.e.m.'s; *P<0.0001; **P<0.001; unpaired *t*-tests. Scale bars, 10 μm. Dashed bar, 2 μm.

To investigate whether a compromised DNA damage checkpoint is a feature of aged oocytes we have induced DNA damage in oocytes from mice >50 weeks of age and examined their ability to progress through MI. We verify previous reports^13^,^38^ that young and aged oocytes progress through and complete MI at similar rates (∼80%) (Fig. 4a). However, after DNA damage, over 50% of oocytes from old mothers progress through MI compared with only 12% of controls (Fig. 4a). We confirmed that Etoposide treatment induces similar levels of DNA damage in prophase-arrested young and old oocytes, thus it appears unlikely that the ability of Etoposide-treated aged oocytes to progress through MI is due to a reduced level of damage (Supplementary Fig. 5). Although, a significantly increased proportion of aged oocytes do progress through MI in the presence of DNA damage, the time course of this progression is delayed by an average of 4 h compared with control young oocytes (Fig. 4b). The DNA-damaged oocytes that reach MII show gross abnormalities with deformed spindle structures (Fig. 4c, Supplementary Fig. 6), scattered spindle chromosomes and cytoplasmic chromatin (Fig. 4c–e), unlike undamaged aged oocytes where, although there is chromosome misalignment^16^,^39^,^40^, the chromosomes tend to be located at the metaphase plate area (Fig. 4c–e). The combination of high rates of MI exit with a delay in the timing of Pb1 extrusion and grossly abnormal MII chromosome configuration resembles that of young Etoposide-treated oocytes under conditions of SAC dysfunction (Fig. 3).

Given that we have shown the SAC is necessary for the DNA damage MI checkpoint, a compromised SAC in aged oocytes may explain the increased rate of MI progression. To test this, we have measured SAC components in Etoposide-treated young and aged oocytes 8 h post GVBD when the SAC in untreated control oocytes is satisfied (Fig. 2c–e). We find that Bub1 shows a 15% decrease and BubR1 a 35% decrease in kinetochore accumulation in Etoposide-treated aged oocytes compared with Etoposide-treated young controls (Fig. 4f–h). We find that Mad2 shows only a small non-significant decrease in aged oocytes (Fig. 4h). We also examined kinetochore localization of Polo-like kinase 1 (Plk1) in these conditions. Although, Plk1 is not considered a SAC component its presence at kinetochores is an indication of an active SAC^41^. Our data show that similar to BubR1, there is an average 38% decrease in Plk1 at kinetochores of Etoposide-treated aged oocytes. (Fig. 4h). This decrease in accumulation of SAC components is also reflected by a similar 30% decrease in CREST fluorescence in Etoposide-treated old oocytes. Reduced CREST staining in aged oocytes has also been observed by other researchers^18^. These studies show that kinetochore localization of key SAC components and regulators are disrupted in Etoposide-treated aged oocytes, offering a likely explanation for why aged oocytes can avoid the DNA damage MI checkpoint.

To determine if the compromised DNA damage checkpoint in aged oocytes is due to a fundamental problem in kinetochore function or if it is exclusive to DNA damage, we have quantified SAC components in control conditions in young and aged oocytes. The data show that a similar decrease in SAC components and CREST labelling is seen in aged oocytes, even in the absence of DNA damage (Supplementary Fig. 7). Therefore, the inability of aged oocytes to launch a robust checkpoint in response to DNA damage in MI is not likely to be caused by inefficient recruitment of SAC components, but due to the low abundance of key SAC proteins and a general compromise in kinetochore function in aged oocytes.

There is some controversy as to whether the SAC is compromised in old oocytes. Initial studies indicated on the basis that young and old oocytes have similar MI kinetics, that the SAC is not compromised^38^. However, other studies have reported that Mad2 levels are reduced on kinetochores of aged oocytes^18^, while others have shown a decrease in mRNA for BubR1 and Plk1 in aged oocytes^40^,^42^. Our data provide further evidence that the SAC is compromised in old oocytes and we offer this as a likely explanation for the inability of aged oocytes to establish an efficient MI checkpoint in response to DNA damage.

Another possible explanation for the muted DDR in aged oocytes is that they have depleted levels of Cohesin^12^,^13^,^15^. In recent years it has been shown that Cohesin has an important role in the DDR^43^,^44^ and has also been implicated in SAC regulation in oocytes^45^. Therefore, we examined whether the absence of Cohesin is sufficient to silence the MI response to DNA damage. We used a knockout (KO) mouse model where the meiosis-specific Cohesin component Smc1β is removed^46^. As in the case of aged mice, the *Smc1β* KO mice show significant loss of chromosome cohesion integrity^47^. We find that *Smc1β* KO oocytes show significantly reduced rates of Pb1 extrusion (49% versus 84% in WT oocytes) and that those that do extrude a Pb1 take an average of 3 h longer to do so (Supplementary Fig. 8a,b) possibly due to SAC activation. Monitoring of Pb1 extrusion in Etoposide-treated *Smc1β* KO and WT oocytes shows that they launch a similar DNA damage MI checkpoint (Supplementary Fig. 8a), albeit somewhat less effective than that seen in experiments described thus far (see Fig. 1). Furthermore, the Etoposide-treated *Smc1β* KO and WT oocytes that do progress through MI achieve this on average 6 h later than untreated WT oocytes (Supplementary Fig. 8b). The similar response of WT and *Smc1β* KO oocytes to DNA damage suggests that loss of Cohesin is not responsible for the ability of aged oocytes to bypass the DNA damage checkpoint in MI.

In summary, genomic instability has been known to result from DNA damage^48^,^49^. More specifically in oocytes, induction of DNA damage in the form of DSBs at the G2/prophase stage can cause abnormalities in chromosome structure^50^,^51^. The implications become greater with age since aged oocytes are more prone to genomic instability^10^,^52^. In the absence of a traditional G2/prophase DNA damage checkpoint, the mammalian oocyte has developed a meiotic M-phase checkpoint to identify DNA damage and prevent the production of mature oocytes that could lead to abnormal embryos. This MI DNA Damage checkpoint is invoked by the SAC and can be modulated by SAC regulators such as MAPK. Therefore, the SAC is the major gatekeeper for oocytes harbouring DNA damage. We find that this gatekeeping function is compromised in oocytes from old mice such that a larger proportion of aged oocytes progress through MI in the presence of DNA damage. The explanation appears to be that the SAC and/or kinetochore function is disrupted in old oocytes, rather than due to loss of Cohesin. The implication is that aged oocytes are not only prone to aneuploidy^10^,^11^,^53^ but also to DNA damage-induced chromosomal instability. This is particularly important in assisted reproduction technologies due to the potential DNA damage that may be caused to oocytes in the laboratory^54^,^55^. Our findings may lead to new insights into the potential causes for the appearance of chromosomal aberrations in the oocytes of women of advanced reproductive age and further explain age-related infertility.

## Methods

### Mouse oocyte collection, culture and micro-injection

Young and aged oocytes were collected from ovaries of 6–8-week old and >50-week old MF1 female mice, respectively, 46–48 h following intraperitoneal administration of 7 international units (IU) of Pregnant mare's serum gonadotropin (PMSG; Intervet). Animal experiments were covered by a Licence from the Home Office (UK). For the generation of *Smc1*β knockout mice a vector for targeting exon 10 of the mouse *Smc1*β gene was designed. The vector was incorporated into W4/129S6 embryonic stem cells (Taconic Inc.), which were then injected into C57BL/6 blastocysts^46^. Cumulus-enclosed GV-stage oocytes were recovered by mechanical perforation of the ovaries with a 27-guage needle. The cumulus cells were removed by repeated pipetting using narrow-bore glass Pasteur pipettes. Oocytes were placed in drops of M2 medium (Sigma) supplemented with 200 μM 3-isobutyl-1-methylxanthine (IBMX; Sigma) under mineral oil (Sigma) on a 37 °C hot block in order to keep the oocytes arrested at the GV stage. Following collection and/or micro-injection, the oocytes were released from IBMX and allowed to undergo oocyte maturation (GVBD and progression through MI) in M16 at 37 °C in a humidified atmosphere of 5% CO_2_ in air. Micro-injection was performed in M2-IBMX^56^. Micropipettes were inserted into oocytes using the negative capacitance overcompensation capabilities of an electrophysiological amplifier (World Precision Instruments). Oocytes were immobilized with a holding pipette (Hunter Scientific). Injection volume was estimated at 2–5% of total oocyte volume by cytoplasmic displacement. Precise injection volume was achieved by the use of a pressure regulator (Pneumatic PicoPump; World Precision Instruments). Micro-injection was used for the introduction of cRNAs and Morpholinos (MOs) into oocytes.

### cRNAs and MO oligonucleotides

For inhibiting the function of Bub1 we used dominant negative Bub1 cRNA prepared from the pEFT7MCS-DnBub1 plasmid (kind gift from Tim Hunt, Cancer Research UK)^57^. For expressing Geminin-GFP we used a pcDNA3.1-Geminin-GFP (kind gift from Zoi Lygerou, University of Patras, Greece)^20^. The cRNAs were prepared from the T7 promoter of the vectors. cRNAs were synthesized and polyadenilated using the mMessage mMachine kit (Ambion), purified in nuclease-free water through Rneasy columns (Quigen) to a concentration of ∼1 μg μl^−1^, aliquoted and stored at −80 °C.

For the inhibition of endogenous protein expression we used MOs (Gene Tools LLC) that are fully characterized in mouse oocytes: Mos MO, 5′-CACAGGCTTAGAGGCGAAGGCATT-3′ (ref. 31); Mad2 MO, 5′-GCTCTCGGGCGAGCTGCTGTGCCAT-3′ (ref. 58). MOs were dissolved in nuclease-free water at a concentration of 2 mM. MO micro-injections were performed 5 h before release from IBMX.

### Treatments

DSBs were performed by treating oocytes with 100 μg ml^−1^ Etoposide (Sigma), 10 μg ml^−1^ Phleomycin (Sigma) or 20 μM Doxorubicin (Sigma) for 1 h at the GV stage. For inhibitor treatments, oocytes were incubated in M16 containing 50 μM of the MAPK kinase inhibitor UO126 (Promega), 2 μM of the Mps1 inhibitor AZ3146 (Santa Cruz Biotechnology, Inc.) and 40 μM of the ATM inhibitor ATMi (Calbiochem). Inhibitors were applied 5 h post GVBD for the duration of the experiment. Nocodazole (Calbiochem) was used at 100 nM following GVBD and for the duration of the experiment.

### Immunofluorescence

For detection of γH2AX and β-tubulin oocytes were fixed in PBS containing 4% paraformaldehyde and then permeabilized in 0.5% Triton X-100. The oocytes were then incubated in blocking buffer (PBS, 3% BSA) followed by incubation with the primary antibodies^59^. For kinetochore immunolabelling, oocytes were fixed and permeabilized in PHEM buffer (60 mM Pipes, 25 mM Hepes, 10 mM EGTA and 2 mM MgCl_2_) containing 4% paraformaldehyde and 0.5% Triton X-100. For labelling we used the primary antibodies: rabbit anti-γH2AX (1/800; 4418-APC; Trevigen), rabbit anti-Mad2 (1/300; PRB-452C; Covance), mouse anti-β-tubulin (1/1000; T4026; Sigma), mouse Anti-Plk1 (1/300; sc-17783, Santa Cruz Biotechnology), human CREST antiserum (1/300; a kind gift from Bill Earnshaw, University of Edinburgh, UK and Greg FitzHarris, University of Montreal, Canada), sheep anti-Bub1 and anti-BubR1 (1/300; kind gift from Stephen Taylor, University of Manchester, UK). The secondary antibodies used were: Alexa Fluor 488-conjugated goat anti-rabbit (1/500; A11008; Invitrogen), Alexa Fluor 488-conjugated goat anti-mouse (1/500; A11029; Invitrogen), Alexa Fluor 488-conjugated donkey anti-sheep (1/500; A11015; Invitrogen) and Alexa Fluor 546-conjugated goat anti-human (1/500; A21089; Invitrogen). For DNA staining, we incubated oocytes with Hoechst 33342 (10 μg ml^−1^; Sigma). Serial *z* sections of fixed oocytes were acquired by the use of a laser-scanning confocal microscope imaging system (LSM 510 META; Carl Zeiss) with the following band pass emission filters: 385–470 nm (Hoechst 33342), 505–530 nm (Alexa Fluor 488) and 585–615 nm (Alexa Fluor 546). *z* sections were analysed and projected into one image using the LSM image browser (Carl Zeiss).

### Epifluorescence imaging

An inverted microscope (Axiovert 100; Carl Zeiss) equipped with an interline cooled CCD camera (Princeton Instruments MicroMAX; Roper Scientific) was used for the recording of epifluorescence images of oocytes incubated in M2 medium at 37 °C. GFP-tagged Geminin was imaged using a filter set comprised of an excitation filter with a band pass of 450–490 nm, a dichroic mirror 510 nm and a band pass 505–520 nm emission filter. A DAPI (4,6-diamidino-2-phenylindole) filter set was used for imaging Hoechst 33342 (excitation filter: 340–380 nm, dichroic mirror: 400–420 nm, emission filter: 435–485 nm). Oocytes were imaged every 15 min to minimize photobleaching and photodamage. Data were collected and analysed using MetaMorph and Meta-Fluor software 6.1 (Molecular Devices).

### Data analysis and statistics

The rate of Geminin-GFP destruction per hour was calculated according to the formula (Fl_1_–Fl_2_)/Fl_1_(*t*_2_−*t*_1_). For the controls, Fl_1_ is the fluorescence just before the start of the metaphase to anaphase destruction phase, while Fl_2_ is the fluorescence at the end of the destruction phase. t_1_ and t_2_ are the time points that correspond to Fl_1_ and Fl_2_, respectively. Background fluorescence was subtracted from cell fluorescence. For Etoposide-treated oocytes, *t*_1_ and *t*_2_ represent hourly time points from 7 to 12 h post GVBD. For kinetochore fluorescence experiments, the oocytes (control versus treated and young versus aged) of all the replicates from the same experiment, for one SAC component, were manipulated under the same conditions and imaged under identical settings. The fluorescence of each kinetochore was measured from a single *z* section. The mean fluorescence of an area surrounding the kinetochore was used as background and was subtracted from kinetochore fluorescence. Kinetochores were considered positive for a SAC component when the fluorescence was detectable after subtracting background. For normalization, we used the values of young oocyte kinetochores. ImageJ software was used for measuring the fluorescence. For colocalization measurements, we used *z-*projection images of CREST and Mad2 or Bub1. An area including all the kinetochores of a cell in the CREST images was used for localization comparison. The extent of colocalization was identified by the Coloc 2 function of ImageJ. We show Pearson's correlation coefficient calculations and values on a scale −1 to +1, where −1 is total negative correlation, 0 is no correlation and +1 is total positive correlation. M1, M2 and Costes coefficients show the same level of colocalization. For the measurement of spindle length, a line was drawn using ImageJ between the fluorescing edges of the spindle where the spindle poles are located. For determining spindle width, a line, perpendicular to the line between the spindle poles, was drawn at the area of the major width of the spindle. Statistical analysis was performed using unpaired *t*-tests in Prism software (GraphPad Software). Analysis was performed for three replicate experiments, unless otherwise stated and represented as means and s.e.m.'s.

## Additional information

**How to cite this article:** Marangos, P. *et al.* DNA damage-induced metaphase I arrest is mediated by the spindle assembly checkpoint and maternal age. *Nat. Commun.* 6:8706 doi: 10.1038/ncomms9706 (2015).

## Supplementary Material

Supplementary Information Supplementary Figures 1-8
