# Supplementary material for: DNA damage-induced metaphase I arrest is mediated by the spindle assembly checkpoint and maternal age
Source: Nat Commun. 2015 Nov 2;6:8706. doi: 10.1038/ncomms9706 (PMC4667640; doi:10.1038/ncomms9706)
Supplement: Supplementary Information — Supplementary Figures 1-8 [file ncomms9706-s1.pdf]

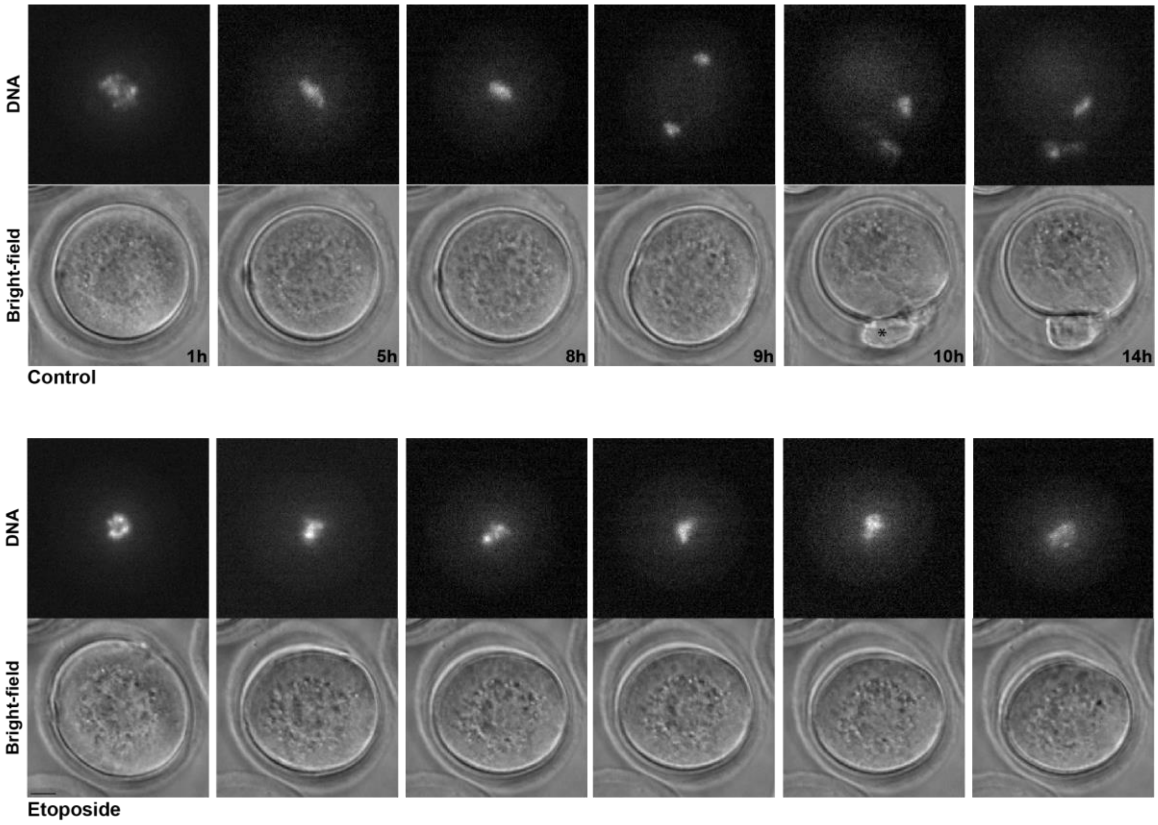

Supplementary Figure 1: DNA damaged oocytes fail to exit MI.

Representative time-lapse fluorescence microscopy images of oocytes undergoing meiotic maturation (MI) in the presence or absence (Control) of Etoposide. GV-stage oocytes were incubated with Etoposide for 1h and then released from IBMX. The asterisk denotes the Pb1 of the Control oocyte. No Pb1 is extruded from the Etoposide-treated oocyte. Hoechst 33342 was used for DNA staining. Time 0h: GVBD. Bar, 10µm. n=2 experiments. ≥ 15 oocytes per group.

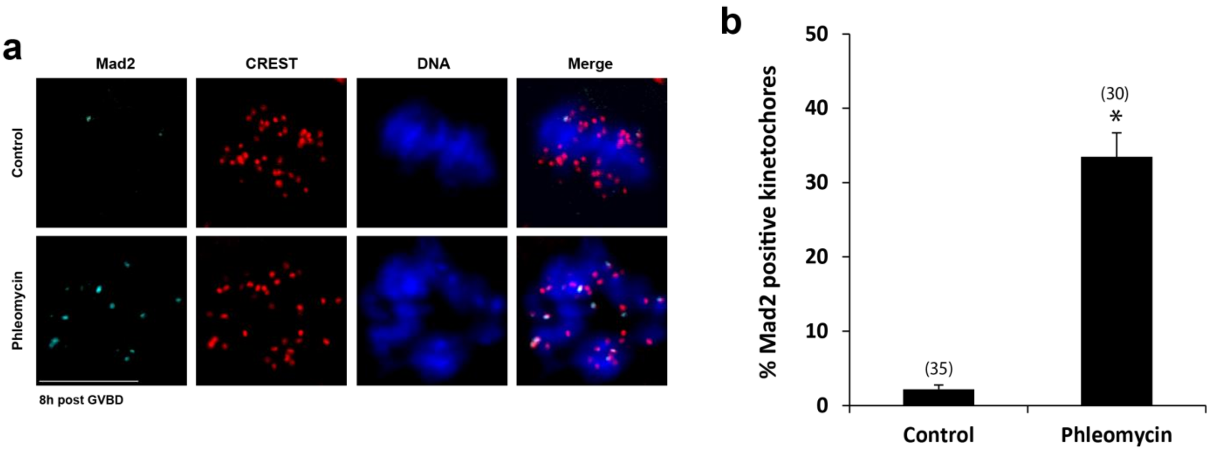

Supplementary Figure 2: Phleomycin-induced MI arrest is caused by SAC activation.

Phleomycin-treated oocytes arrested in MI are Mad2 positive. **(a)** Representative z-projection images of immunostaining for CREST and Mad2 in non-treated controls and oocytes treated with Phleomycin. Oocytes were fixed 8h post GVBD. **(b)** Proportion of Mad2 positive kinetochores 8h post GVBD. Data analysed from experiments shown in **a**. The total number of cells measured is shown in parentheses. *n*=3 experiments. Bars, 10μm. Data are represented as mean. Error bars show SEMs. *, P<0.0001; unpaired t-test.

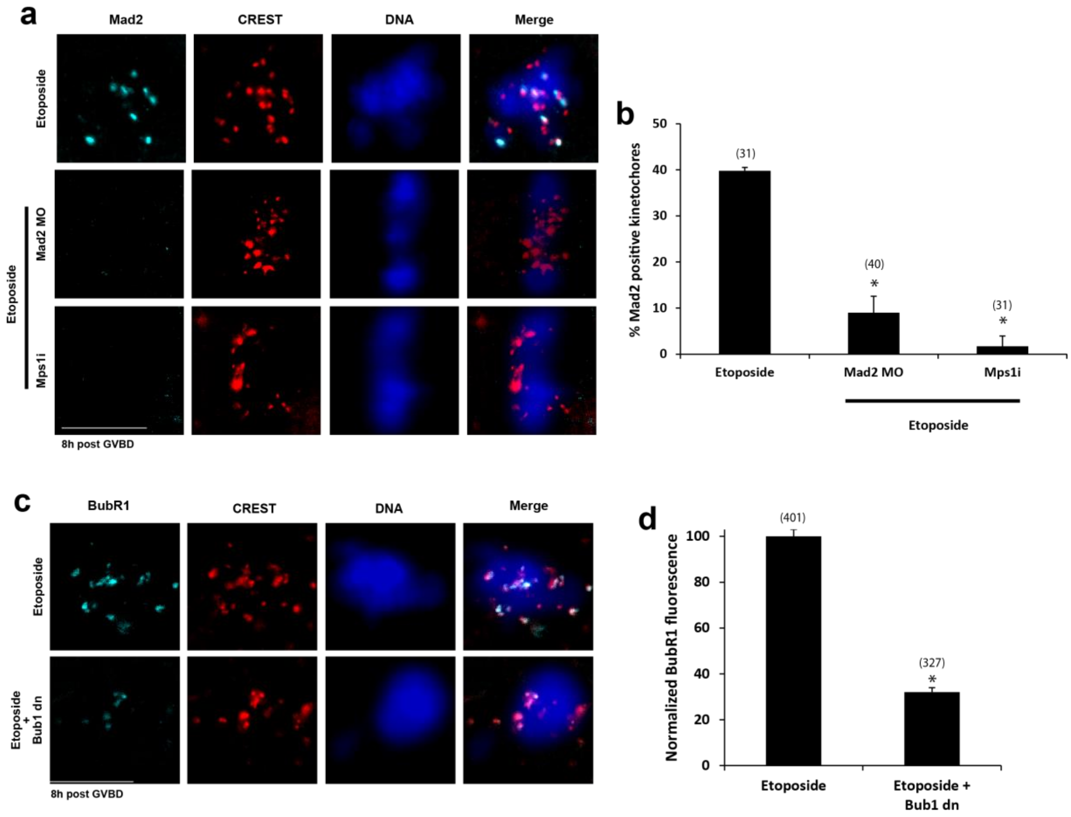

Supplementary Figure 3: Mad2 MO, Mps1i and Bub1 dn cause the dissociation of SAC components from MI kinetochores.

(a,b) Depletion of Mad2 (Mad2 MO) or inhibition of Mps1 (Mps1i) in Etoposide-treated oocytes cause the dissociation of Mad2 from kinetochores. Oocytes were treated as in Figure 3. **(a)** Representative z-projection images of immunostaining for Mad2 and CREST in oocytes treated with Etoposide. Oocytes were fixed 8h post GVBD. **(b)** Proportion of Mad2 positive kinetochores 8h post GVBD. Data analysed from experiments shown in **a**. The total number of cells measured is shown in parentheses. *n*=3 experiments. **(c,d)** Injection of Bub1dn prior to Etoposide treatment leads to significantly reduced localization of BubR1 on kinetochores. **(c)** Representative z-projection images of immunostaining for BubR1 and CREST in oocytes treated with Etoposide. Oocytes were fixed 8h post GVBD. **(d)** BubR1:CREST ratios were determined and normalised to the intensity of non-injected Etoposide-treated oocytes. The total number of kinetochores measured is shown in parentheses. Bars, 10μm. Data are represented as mean. Error bars show SEMs. *, P<0.0001; unpaired t-test.

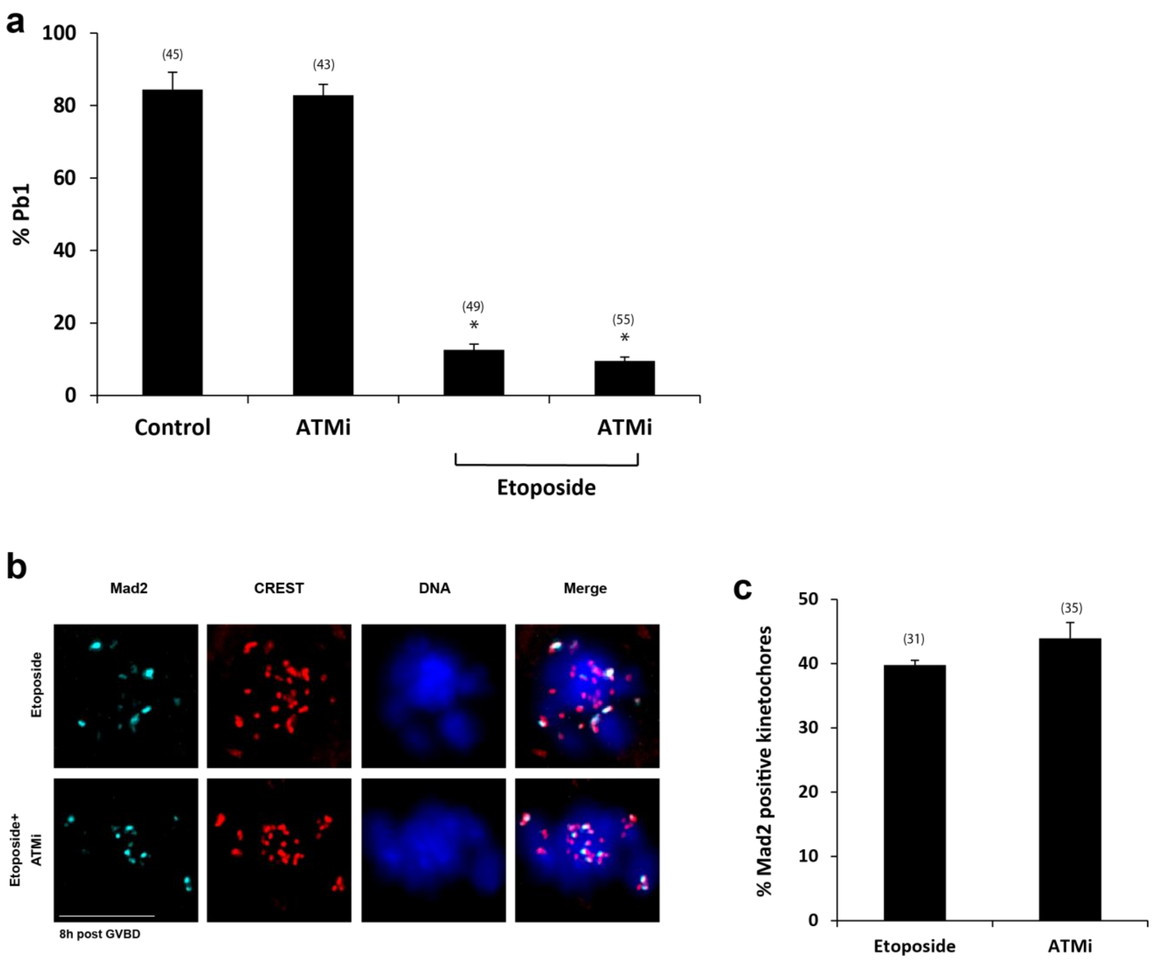

Supplementary Figure 4: The DNA damage response factor ATM does not participate in the SAC-dependent MI arrest following DNA damage.

Control and DNA-damaged MI oocytes were treated with an inhibitor for ATM (ATMi) 5h post GVBD for the duration of the experiment. **(a)** ATM inhibition does not alleviate DNA damage-induced MI arrest. Pb1 extrusion was scored 18h after release from IBMX and oocytes continued to be monitored for Pb1 extrusion until at least 24h from IBMX release. **(b,c)** Mad2 localization at the kinetochores of Etoposide-treated oocytes is not perturbed following inhibition of ATM. **(b)** Representative z-projection images of immunostaining for Mad2 and CREST in oocytes treated with Etoposide and ATMi or without ATMi. Oocytes were fixed 8h post GVBD. **(c)** Proportion of Mad2 positive kinetochores 8h post GVBD. Data analysed from experiments shown in **a**. The total number of oocytes examined in at least 3 experiments is

shown in parentheses. Bars, 10µm. Data are represented as mean. Error bars show SEMs. *, P<0.0001; unpaired t-test.

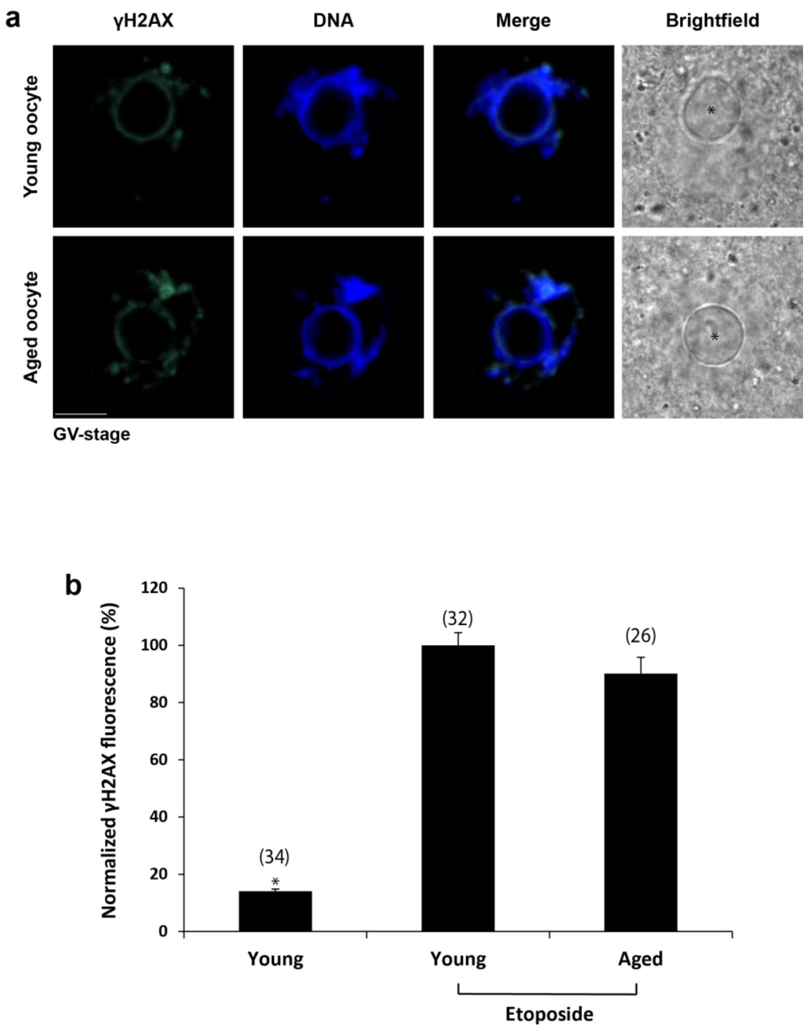

Supplementary Figure 5: Young and aged oocytes respond in the same way to DSB-inducing agents.

(a) Young and aged oocytes bear the same level of DNA damage following exposure to Etoposide. Representative images of γH2AX and DNA labelling in young and aged oocyte nuclei. The oocytes were fixed at the GV-stage following 1h of treatment with 100µg/ml Etoposide. The asterisk denotes the nucleolus. Bars, 10µm. **(b)** Quantification of γH2AX fluorescence of oocytes represented in **a**. Fluorescence was normalized to the intensity of young oocytes. The DNA area, identified by Hoechst 33342, was used for γH2AX measurements. Cytoplasmic fluorescence was used as background and subtracted from DNA fluorescence. Young and aged oocytes were collected and manipulated on the

same day and imaged under the same conditions. The total number of oocytes examined is shown in parentheses. *n*≥3 experiments. Data are represented as mean. Error bars show SEMs. *, P<0.0001; unpaired t-test.

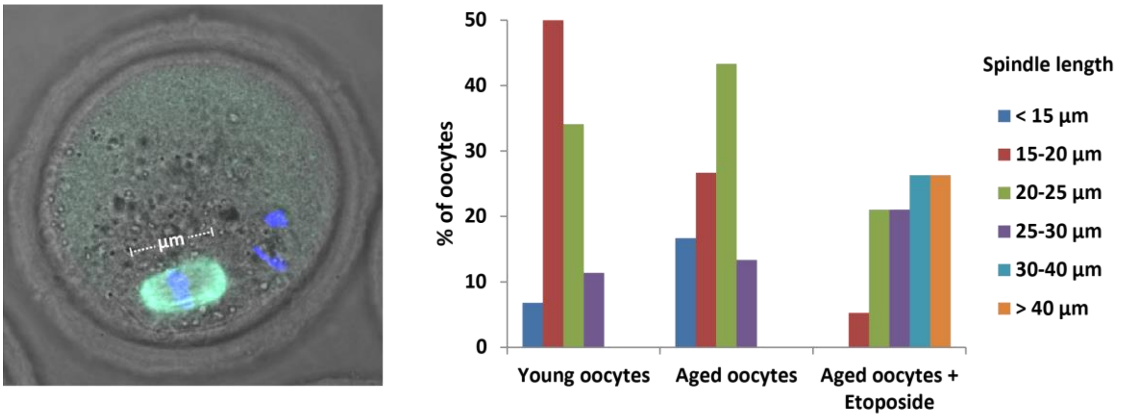

Supplementary Figure 6: Spindle length in MII oocytes.

Aged Etoposide-treated oocytes possess deformed spindle structures with length usually longer than young untreated oocytes. MII oocytes of Figure **4c** where used to calculated spindle length. **(a)** For the measurement of spindle length, a line was drawn between the fluorescing edges of the spindle where the spindle poles are located. **(b)** Spindle length analysis shows that at least half of DNA damaged aged oocytes possess spindle structures much longer (>30 µm) than control MII oocytes (15-25 µm).

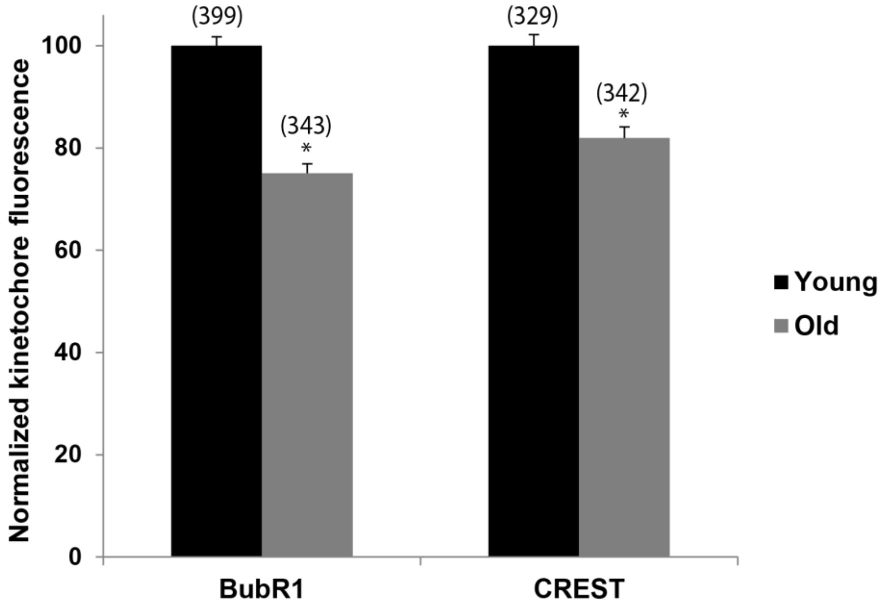

Supplementary Figure 7: Aged oocytes show weaker SAC and kinetochore component accumulation than young oocytes.

Quantification of BubR1 and CREST kinetochore fluorescence of MI oocytes. The oocytes were fixed 6h after release from IBMX and immunostained for BubR1 and CREST. BubR1 and CREST show weaker staining in aged oocytes. Kinetochore fluorescence was normalized to the intensity of young oocytes. Young and aged oocytes were manipulated and imaged under the same conditions and identical imaging settings. The number of kinetochores measured is shown in parentheses. n=2 experiments. Data are represented as mean. Error bars show SEMs. *, P<0.0001; unpaired t-test.

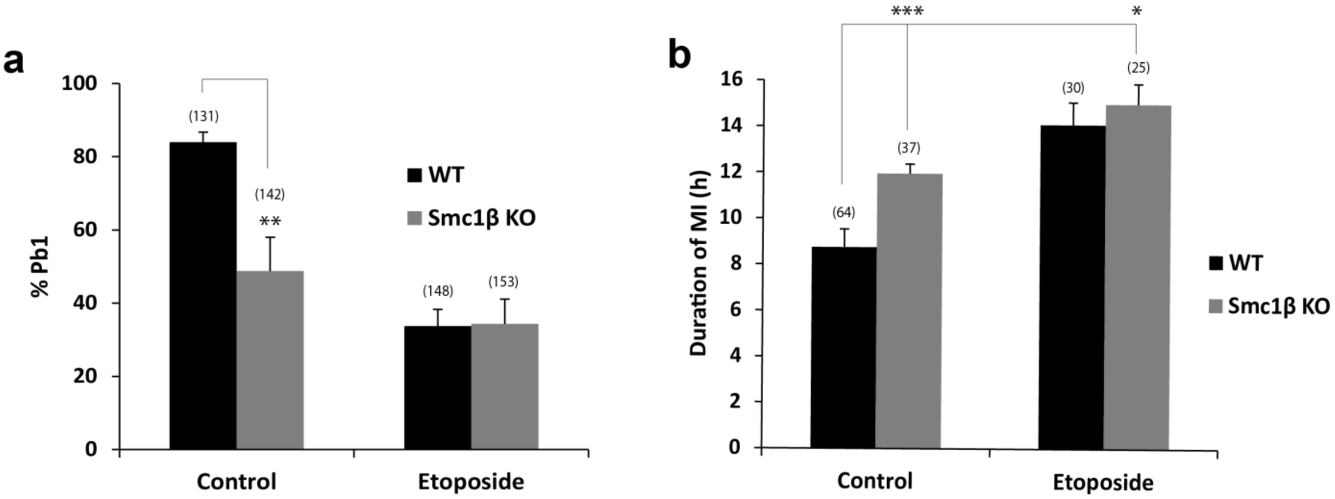

Supplementary Figure 8: Failure of MI arrest in response to DNA damage in aged oocytes is independent of Cohesin loss.

(a) Rates of Pb1 extrusion in WT and Smc1β KO oocytes. Oocytes were monitored for Pb1 extrusion 18h after release from IBMX. WT and Smc1β KO oocytes were collected and manipulated on the same day under the same conditions. The total number of oocytes examined is shown in parentheses. *n*=7 experiments. Note that Smc1β KO oocytes show lower levels of Pb1 extrusion with and without treatment with Etoposide. **(b)** WT and Smc1β KO oocytes were subjected simultaneously to time-lapse microscopy for identifying the timing of Pb1 extrusion and determining the duration of MI (GVBD to Pb1 extrusion). Pb1 extrusion is delayed significantly in Smc1β KO oocytes. The total number of oocytes examined is shown in parentheses. *n*=4 experiments. Data are represented as mean. Error bars show SEMs. *, P<0.0001; **, P<0.001; ***, P<0.005; unpaired t-tests.
